# Sensitivity of different MRI sequences in the early detection of melanoma brain metastases

**DOI:** 10.1371/journal.pone.0193946

**Published:** 2018-03-29

**Authors:** Katerina Deike-Hofmann, Daniel Thünemann, Michael O. Breckwoldt, Daniel Schwarz, Alexander Radbruch, Alexander Enk, Martin Bendszus, Jessica Hassel, Heinz-Peter Schlemmer, Philipp Bäumer

**Affiliations:** 1 Department of Radiology, German Cancer Research Center, DKFZ, Heidelberg, Germany; 2 Department of Neuroradiology, University of Heidelberg Medical Center, Heidelberg, Germany; 3 Department of Dermatology, National Center for Tumor Diseases, NCT, University of Heidelberg Medical Center Heidelberg, Germany; University of Queensland Diamantina Institute, AUSTRALIA

## Abstract

**Background:**

After the emergence of new MRI techniques such as susceptibility- and diffusion-weighted imaging (SWI and DWI) and because of specific imaging characteristics of melanoma brain metastases (MBM), it is unclear which MRI sequences are most beneficial for detection of MBM. This study was performed to investigate the sensitivity of six clinical MRI sequences in the early detection of MBM.

**Methods:**

Medical records of all melanoma patients referred to our center between November 2005 and December 2016 were reviewed for presence of MBM. Analysis encompassed six MRI sequences at the time of initial diagnosis of first or new MBM, including non-enhanced T1-weighted (T1w), contrast-enhanced T1w (ceT1w), T2-weighted (T2w), T2w-FLAIR, susceptibility-weighted (SWI) and diffusion-weighted (DWI) MRI. Each lesion was rated with respect to its conspicuity (score from 0—not detectable to 3—clearly visible).

**Results:**

Of 1210 patients, 217 with MBM were included in the analysis and up to 5 lesions per patient were evaluated. A total of 720 metastases were assessed and all six sequences were available for 425 MBM. Sensitivity (conspicuity ≥2) was 99.7% for ceT1w, 77.0% for FLAIR, 64.7% for SWI, 61.0% for T2w, 56.7% for T1w, and 48.4% for DWI. Thirty-one (7.3%) of 425 lesions were only detectable by ceT1w but no other sequence.

**Conclusions:**

Contrast-enhanced T1-weighting is more sensitive than all other sequences for detection of MBM. Disruption of the blood-brain-barrier is consistently an earlier sign in MBM than perifocal edema, signal loss on SWI or diffusion restriction.

## Introduction

Brain metastases are the most frequent brain tumors[1], most often caused by breast cancer, lung cancer, and by melanoma[2]. In the assessment of patients with cerebral metastases, MR imaging is the most sensitive diagnostic technique and an essential part of the follow-up in patients with metastatic melanoma. Approximately 40–50% of stage IV melanoma patients develop clinical manifestations of melanoma brain metastasis (MBM)[3,4], while autopsy studies suggest an even higher prevalence of 55–75%[3,5,6].

Since the introduction of new therapeutic possibilities in melanoma[7–12], early detection of metastases has an increasingly important role in managing patients with MBM since their detection could be crucial for timely change of therapy.

The imaging hallmark of brain metastases has been contrast enhancement[13–16]. Previous imaging studies on MBM have additionally focused on MRI signal intensity characteristics, e.g. high signal intensity relative to cortex on unenhanced T1-weighted (T1w) images[17,18] as well as T2*-weighted signal intensity loss[19]. Both of these imaging findings could conceivably be caused by intratumoral melanin content as well as hemorrhage[17–20]. Given the high sensitivity of SWI for susceptibility artifacts and their frequent presence in MBM, it is conceivable that SWI might be the most sensitive technique for detection of at least a subset of MBM.

Further, an increasing number of patients and also referring physicians have become reluctant in accepting gadolinium based contrast agents due to their potential retention in the brain[21,22], impairment of renal function or possible contrast agent allergy and would prefer non contrast agent examinations, especially given the frequency of screening cranial MRI (cMRI) at stage IIIC and IV of melanoma.

It is therefore a relevant and hitherto unresolved issue which MRI sequence allows for reliable, early detection of new MBM and whether contrast administration must be considered mandatory. The aim of this study was to identify the MRI sequences with highest diagnostic sensitivity for reliable early detection of MBM.

## Materials and methods

### Patients and ethics

This retrospective study was approved and patients´ consent was waived by the ethics committee of the University of Heidelberg. We reviewed the medical record and neuroimaging studies of all patients who were referred to our imaging center between November 2005 and December 2016 with a diagnosis of melanoma. Patients with brain metastasis were included in the study and cranial MRIs (cMRI) at the time of first diagnosis of new MBM (hereinafter referred to as first diagnosis MRI) were analyzed. Patients without available cMRI at initial diagnosis had to be excluded.

### Imaging protocol

The majority of cMRI scans were performed at our institution (72.0%, 223/310) on a Siemens Magnetom Symphony 1.5 Tesla MRI system (67.3%, 150/223). For detailed information of the used MRI systems and contrast media see Table 1.

**Table 1:** MRI systems and contrast media.

MRI unit				Contrast media			
name	B [T]	Internally n = 223	Externally n = 87	name	c [mol/L]	Internally n = 223	Externally n = 87
S Avanto	1.5	33 (14.8%)	11 (12.6%)	MultiHance®	0.5	173 (77.6%)	0 (0.0%)
S Symphony	1.5	150 (67.3%)	13 (14.9%)	Magnevist®	0.5	10 (4.5%)	4 (4.6%)
S Aera	1.5	3 (1.3%)	5 (5.7%)	Dotarem®	0.5	18 (8.1%)	21 (24.1%)
S Biograph	3	16 (7.2%)	0 (0.0%)	Gadovist®	1	12 (5.4%)	34 (39.1%)
S Prisma Fit	3	1 (0.4%)	1 (1.1%)	Omniscan®		1 (0.4%)	0 (0.0%)
S Trio Tim	3	20 (9.0%)	21 (24.1%)	Native	-	0 (0.0%)	1 (1.1%)
other	-	0 (0.0%)	36 (41.4%)	NA	NA	9 (4.0%)	26 (29.9%)

**Abbr.:** S, Siemens; B, magnetic field strength; T, Tesla; intern, cranial MRI (cMRI) was performed at the German Cancer Research Center (DKFZ); extern, cMRI was performed at an institution other than the DKFZ; c, molar concentration.

The following sequences were analyzed: non-enhanced T1-weighted (T1w), contrast-enhanced T1-weighted (ceT1w), T2-weighted (T2w), T2-weighted fluid-attenuated inversion recovery (FLAIR), susceptibility-weighted (SWI), and diffusion-weighted (DWI) imaging (for the MR protocol see Table 2).

**Table 2:** Siemens Magnetom Symphony 1.5 Tesla MR protocol.

	T1w	T2w	ceT1w	SWI	FLAIR	DWI
TE [ms]	12	101	17	40/21	98	91
TR [ms]	500	5200	525	49/1200	8000	3500
ST [mm]	4	4	4	1.9/3	5	5
B-value [s/mm^2^]	-	-	-	-	-	800
TI [ms]	-	-	-	-	2340	-
No. of Averages	1	2	1	1	2	1
Acquisition Time [min:sec]	4:00	3:53	4:00	5:40	5:38	2:32
Field of View [mm]	230	230	230	240	230	255
No. of slices	37	37	37	80	30	24
Flip angle [°]	90	150	90	15	150	90
Acquisition Matrix	0/320/234/0	0/320/205/0	0/320/234/0	0/320/213/0	0/320/205/0	0/192/192/0

**Abbr.:** T1w, non-enhanced T1-weighted; T2w, T2-weighted; ceT1w, contrast-enhanced T1-weighted; SWI, susceptibility-weighted imaging; FLAIR, fluid-attenuated inversion recovery; DWI, diffusion-weighted imaging (TRACE); TE, Echo Time; TR, Repetition Time; TI, Inversion Time; ST, Slice Thickness.

At our institution, most commonly the contrast agent Gd-BOPTA was administered (173/223, 77.6%, MultiHance®, 0.5 M, Bracco Imaging). All contrast media was manually injected intravenously with a standard dose of 0.1ml/kg for 1 M (Gadovist®) and 0.2ml/kg for 0.5 M contrast agents (Multihance®, Magnevist®, Dotarem®) followed by a saline flush of 20ml. Mean time-to-acquisition amounted 6:08 minutes.

### Image analysis

**Fig 1:**
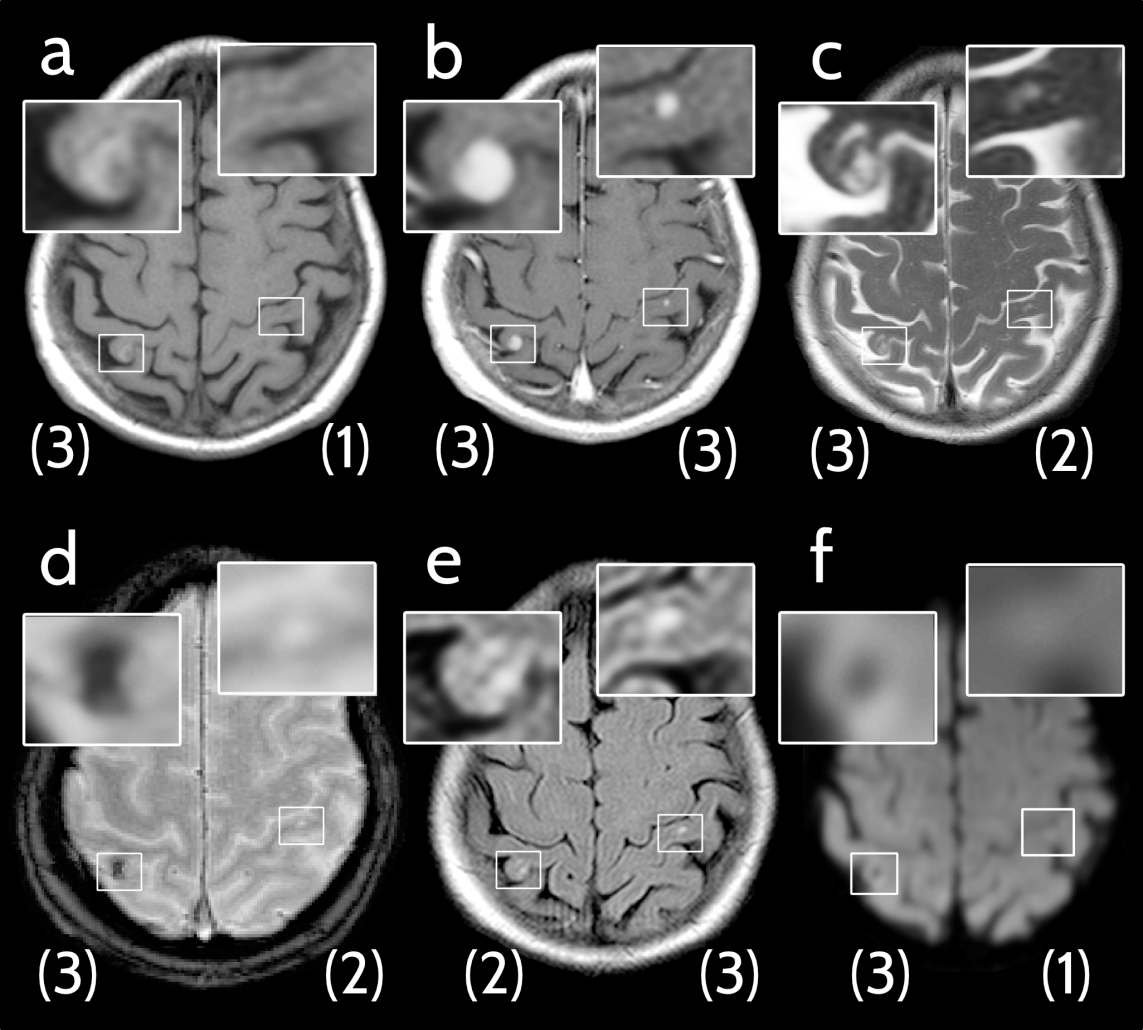
Example of applied image analysis. Axial cMRI scans of a 70-year-old woman with newly diagnosed brain metastases from malignant melanoma (MBM). Conspicuity scores of each MBM are indicated in brackets below the respective lesion. **(a)** Non-enhanced T1-weighted image, right MBM: conspicuity score (CS) 3, left MBM: CS 1. **(b)** Contrast-enhanced T1-weighted image, right MBM: CS 3, left MBM: CS 3. **(c)** T2-weighted image, right MBM: CS 3, left MBM: CS 2. **(d)** Fast low angle shot 2D susceptibility-weighted imaging (SWI), right MBM: CS 3, left MBM: CS 2. **(e)** Fluid-attenuated inversion recovery (FLAIR) image, right MBM: CS 2, left MBM: CS 3. **(f)** Diffusion-weighted image (DWI TRACE), right MBM: CS 3, left MBM: CS 1.

Image analysis was performed on a per-patient and per-lesion basis, with a maximum of five lesions per patient. If a patient had more than five newly developed MBM, the five smallest MBM were analyzed. If available, MRI examinations prior to the first diagnosis MRI (pre-diagnosis MRI) were consulted to evaluate on which sequences MBM were retrospectively detectable at very early stages. Reading of the MBM was performed by one of two radiologists with eight or one year of experience in neuroimaging on our picture archiving and communication system (Centricity PACS, GE Healthcare Integrated IT Solutions, Barrington, Illinois). A pre-reading session was conducted to reach agreement between readers with regard to the different Conspicuity Scores (CS). Sequence order of analysis was random. Image analysis was carried out with readers blinded to the patients’ data and according to the following description:

- In each patient, up to five lesions were rated with regard to lesion conspicuity (4 point ordinal scale: CS = 0, not detectable; CS = 1, pathological correlate detectable with prior knowledge of existence and localization; CS = 2, detectable, CS = 3, clearly visible, see Fig 1).
- Additionally, lesion size for each lesion was determined (product of the longest and the orthogonal diameter) and signal intensity on non-enhanced T1-weighted and susceptibility-weighted imaging was evaluated relative to the contralateral cortex (hyper-, iso-, or hypointense).
- Those MBM with an imaging appearance compatible with leptomeningeal or pachymeningeal localization were also recorded.

### Reference standard

In every patient the diagnosis of the primary tumor of malignant melanoma was confirmed by histopathology. Final diagnosis of MBM for every brain lesion was based on comparison with prior examinations, histopathological data, and most importantly by clinical and MRI follow-up.

### Statistical analysis

**Fig 2:**
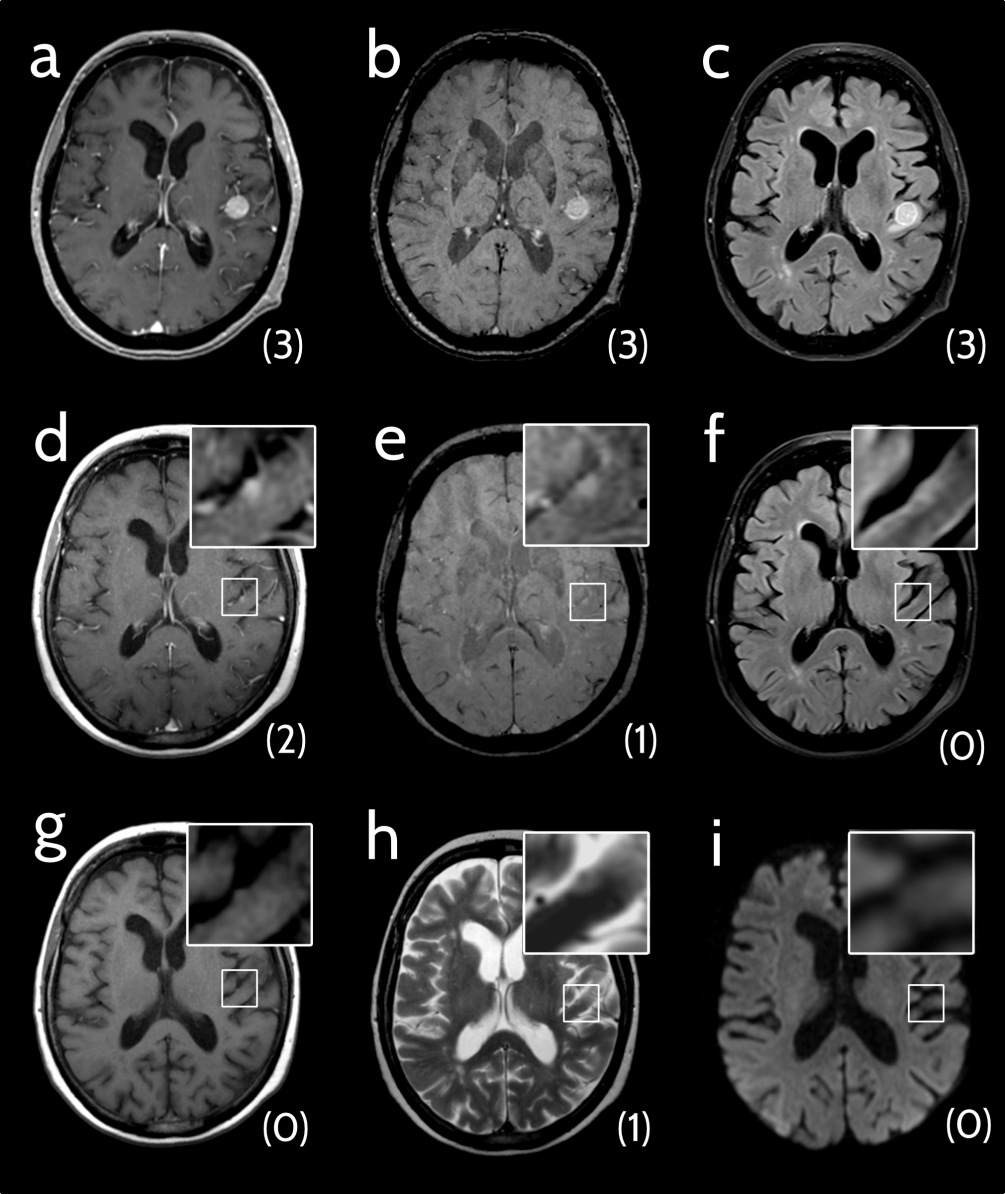
Analysis of pre-diagnosis cMRIs. 69-year-old woman with a melanoma brain metastasis (MBM) that was detectable in an examination prior to the one that led to diagnosis. Conspicuity scores are indicated in brackets below the lesions. **(a-c)** Axial cMRI scans of a patient with newly diagnosed MBM. **(a)** Contrast-enhanced T1-weighted image (ceT1w): conspicuity score (CS) 3. **(b)** Susceptibility-weighted image (SWI), CS 3. **(c)** Fluid-attenuated inversion recovery (FLAIR) image: CS 3. **(d-i)** Axial cMRI scans of the pre-examination (ca. 9 weeks earlier).**(d)** CeT1w: CS 2. **(e)** SWI: CS 1. **(f)** FLAIR: CS 0. **(g)** Non-enhanced T1-weighted image: CS 0. **(h)** T2-weighted image: CS 1. **(i)** Diffusion-weighted image (TRACE): CS 0.

Descriptive statistics were carried out using Microsoft Excel 2016. The mean CS with standard deviation (SD) was calculated and a subgroup analysis was performed for:

- MBM of melanotic and amelanotic primary melanoma,
- MBM with lesion size up to 5 mm in diameter,
- lesions found in the MRI scan prior to the one that led to diagnosis (see Fig 2), and
- MBM sited lepto- or pachymeningeally.

Statistical testing was performed with the software package R (R Foundation for Statistical Computing, version 3.1.1, 2014-07-10). Shapiro-Wilk test was used to test for standard distribution of the data and Wilcoxon signed-rank test was performed to compare the CS of the two most sensitive sequences. Significance was set at the p < 0.05 level.

## Results

### Patient data

Overall, 224 of 1210 (18.5%) melanoma patients had a record of metastatic brain lesions. Seven of these had to be excluded due to unavailable cMRI. Of the remaining 217 patients, 73 were female (33.6%) and 144 male (66.4%). The patients’ mean age (SD) was 59.2 ± 13.1 years. Forty-eight patients (22.1%) displayed a solitary MBM, 81 (37.3%) had 2–4 MBM and 88 patients (40.5%) showed 5 or more lesions.

In total, 720 MBM were evaluated. Median lesion diameter (interquartile range (IQR)) at the time of first diagnosis was 5 mm (3–8 mm). Out of 720 MBM, 77 had an imaging appearance compatible with leptomeningeal (10.7%) and 19 with pachymeningeal (2.6%) localization. No patient depicted a gyriform leptomeningeal enhancement typical for meningiosis[23].

### Conspicuity score (CS) and sensitivity

**Fig 3:**
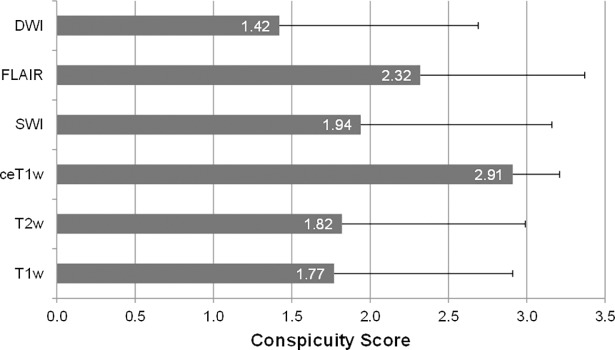
Mean Conspicuity scores and standard deviations of the six different MRI sequences at first diagnosis MRI. Abbr.: DWI, Diffusion-weighted image; FLAIR, Fluid-attenuated inversion recovery; SWI, Susceptibility-weighted image; ceT1w, contrast-enhanced T1-weighted image; T2w, T2-weighted image; T1w, native T1-weighted image.

The mean CS (SD) of all lesions at first diagnosis (n = 720) was 2.9 (±0.3) on ceT1w, 2.3 (±1.1) on FLAIR, 1.9 (±1.2) on SWI, 1.8 (±1.2) on T2w, 1.8 (±1.1) on T1w, and 1.4 (±1.3) on DWI (see Fig 3).

The ceT1w sequence had a significantly higher CS than the second best sequence (paired Wilcoxon-test, *p*-value < 0.001). Notably, not a single metastasis (0/712) was rated with a CS of 0 on ceT1w images, whereas 72 of 673 (10.7%), 124 of 699 (17.7%), 143 of 706 (20.3%), 104 of 496 (21.0%), and 191 of 510 (37.5%) of all lesions had no imaging correlate at all (CS 0) on FLAIR, T1w, T2w, SWI and DWI, respectively. Table 3 shows detailed CS results.

**Table 3:** Conspicuity scores.

	T1w	T2w	ceT1w	SWI	FLAIR	DWI
**Lesions at first diagnosis MRI**						
n of 720	699	706	712	496	673	510
mean CS	1.77	1.82	2.91	1.94	2.32	1.42
±SD	1.14	1.17	0.30	1.22	1.05	1.27
n(CS 0)	124 (17.7%)	143 (20.3%)	0 (0%)	104 (21.0%)	72 (10.7%)	191 (37.5%)
n(CS 1)	179 (25.6%)	132 (18.7%)	2 (0.3%)	71 (14.3%)	83 (12.3%)	72 (14.1%)
n(CS 2)	131 (18.7%)	143 (20.3%)	60 (8.4%)	72 (14.5%)	75 (11.1%)	91 (17.8%)
n(CS 3)	265 (37.9%)	288 (40.8%)	650 (91.3%)	249 (50.2%)	443 (65.8%)	156 (30.6%)
**Lesions at pre-diagnosis MRI**						
n of 33	33	33	33	23	32	24
mean CS	0.48	0.18	1.82	0.39	0.56	0.17
±SD	0.83	0.64	0.88	0.94	1.01	0.64
n(CS 0)	22 (66.7%)	30 (90.9%)	0 (0%)	19 (82.6%)	22 (68.8%)	22 (91.7%)
n(CS 1)	8 (24.2%)	1 (3.0%)	16 (48.5%)	1 (4.3%)	6 (18.8%)	1 (4.2%)
n(CS 2)	1 (3.0%)	1 (3.0%)	7 (21.2%)	1 (4.3%)	0 (0%)	0 (0%)
n(CS 3)	2 (6.1%)	1 (3.0%)	10 (30.3%)	2 (8.7%)	4 (12.5%)	1 (4.2%)
**Lesions ≤ 5 mm diameter at first diagnosis MRI**						
n	385	390	391	270	377	293
mean CS	1.33	1.2	2.72	1.46	1.77	0.83
±SD	1.15	1.11	0.56	1.29	1.22	1.1
**Leptomeningeal lesions**						
n of 720	77	79	76	70	77	63
mean CS	1.84	1.29	2.82	1.33	2.17	0.87
±SD	1.12	1.15	0.48	1.34	1.12	1.08
**Pachymeningeal lesions**						
n of 720	18	19	19	17	19	17
mean CS	2.06	2.11	3.00	2.35	2.58	1.82
±SD	1.00	1.05	0.00	1.06	0.90	1.19
**Amelanotic lesions**						
n of 49	47	48	47	28	44	25
mean CS	1.44	1.98	2.90	2.30	2.53	1.65
±SD	1.21	1.00	0.31	0.97	0.94	1.27
**Melanotic lesions**						
n of 169	159	167	164	130	154	124
mean CS	1.84	1.63	2.88	1.98	2.17	1.48
±SD	1.13	1.22	0.32	1.23	1.13	1.30

**Abbr.:** n, number; CS, conspicuity score; SD, standard deviation; T1w, non-enhanced T1-weighted; T2w, T2-weighted; ceT1w, contrast-enhanced T1-weighted; SWI, susceptibility-weighted imaging; FLAIR, fluid-attenuated inversion recovery; DWI, diffusion-weighted imaging. Total number of lesions with confirmed melanotic / amelanotic primary tumor histology was 218/720 lesions overall.

Since lesions with a CS of 2 and 3 are generally detectable by attentive radiological reading, the sensitivity in the 720 lesions of our patient group was 99.7% (710/712) for ceT1w, 77.0% (518/673) for FLAIR, 64.7% (321/496) for SWI, 61.0% (431/706) for T2w, 56.7% (396/699) for T1w, and 48.4% (247/510) for DWI.

In a total of 425 lesions, all six sequences were available. Without application of contrast agent, 31 of these 425 lesions (7.3%) would not have been detected (CS ≥2 on ceT1w and CS ≤1 on all other sequences).

### Relevance of sequences in addition to contrast-enhanced imaging

In 24 of 712 (3.4%) lesions, at least one sequence was rated with a higher CS than the ceT1w sequence. Eleven of these lesions were displayed more clearly by one other sequence, while in 13 lesions there were multiple superior sequences. In total, SWI was 17 times superior to ceT1w, FLAIR 10 times, T1w 6 times and T2w as well as DWI 4 times each.

The two of 712 lesions that were detectable on ceT1w only with prior knowledge (corresponding to a CS of 1) were better depicted on SWI (CS 3) in one case, and on FLAIR (CS 3) in the other.

### Assessment of pre-diagnosis cMRIs

A pre-diagnosis MRI was available for 456 of 720 lesions (63.3%). These pre-diagnosis MRIs were assessed regarding the presence of MBM with knowledge of the MBM in the first diagnosis MRI. The average time between first diagnosis MRI and pre-diagnosis MRI was 12.3 weeks (±4.2 weeks). Retrospectively, 33 MBM already had a correlate in the pre-diagnosis cMRI (an example is shown in Fig 2), with 18 well detectable (CS ≥2 in any sequence) and the others only detectable retrospectively (CS ≤1 in any sequence). All of these lesions (33/33) had a correlate on ceT1w (100%). In no case (0/33) was another sequence superior to contrast-enhanced imaging in depicting the MBM. Out of the 18 lesions with a CS ≥2 on contrast-enhanced MRI (18/33, 54.5%), 3 were also detectable on SWI (3/23; 13.0%) and 4 on FLAIR (4/32, 12.5%).

## Discussion

As a principal finding, our results demonstrate the highest sensitivity of contrast-enhanced T1-weighted imaging for the detection of brain metastases from malignant melanoma beyond all other sequences. While the usefulness of contrast-enhanced sequences for brain metastases was never questioned, the clear superiority of ceT1w over all other sequences and its exquisite sensitivity was not expected to this degree and has implications for differential diagnosis of unclear suspicious findings in melanoma patients.

It is well known that MRI contrast agents are an essential aid in the early detection of intracranial metastases[24–26]. Nevertheless, it has been assumed that MBM may behave differently. Gaviani et al. found hypointense lesions on T2*-weighted images without correlates on ceT1w images and other unenhanced sequences in patients with malignant melanoma, whereas those lesions in a comparison group with patients with lung cancer were not found. The authors hypothesized that in patients with malignant melanoma those lesions could represent early stages of melanoma metastases[20]. Our results are in contrast to this hypothesis and confirm results of Gramsch et al. who showed that 29 cases of isolated focal areas of susceptibility artifacts in brain parenchyma of 18 melanoma patients were not found to develop as metastases in their follow-up period of 6–46 months[27]. Although approaches similar to ours have been described for metastases of other locations and for different investigations so far[28–32], to our knowledge, our study is the first to compare the visibility of brain metastases across commonly used MRI sequences.

Different MRI sequences reflect different aspects of tumor pathophysiology. Signal characteristics are highly variable across metastases and tumor entities and also change in the progression of the disease. In many higher-grade primary central nervous system tumors, signal increase in T2-weighted and FLAIR images or diffusion restriction most frequently indicate initial diagnosis or tumor progression[33]. In contrast, melanoma brain metastases apparently generally first manifest with disruption of the blood-brain-barrier before signal or diffusion abnormalities due to high cellularity or perilesional edema are detectable.

The high sensitivity of contrast-enhanced imaging for detection of MBM in our study group is supported by three main findings. First, nearly all lesions were conspicuous on ceT1w at the time of first diagnosis. All MBM had a ceT1w correlate, and only two of 712 lesions were detectable in ceT1w only with prior knowledge. Second, only 24 of 712 lesions were considered more conspicuous in a sequence other than ceT1w on the MRI of first diagnosis. And third, only contrast-enhanced imaging depicted a correlate for all lesions that were retrospectively detectable on the pre-diagnosis MRI. Taken together, we believe that these results establish contrast enhancement as the most sensitive early sign for MBM.

This is of considerable importance, because many patients and physicians are increasingly critical of serial injections of gadolinium based contrast agents (GBCAs) due to the unclear effects of the intracerebral deposition of some GBCAs as well as potential contrast agent allergy or renal impairment.

Our study has limitations. First, only intracerebral metastases were assessed. Extracerebral metastases, such as bone metastases of the skull, were not included in the formal analysis even though they are similarly important for staging or therapy decisions. Second, an observer-expectancy bias cannot be precluded, because the ceT1w scan quickly turned out to be superior compared with the other sequences. From then on, the identification of potential lesions might have been guided by the contrast-enhanced scan. Third, only the sensitivity of different sequences in depicting melanoma brain metastases was assessed, whereas the specificity could not be assessed. Moreover, the potential impact of previous therapies such as immuno- or targeted therapies as well as cranial radiotherapy on the depiction of MBM on the different sequences was not evaluated. With FLAIR hyperintensities and susceptibility artifacts being frequent but unspecific findings in the general population and even more so in treated patients, contrast-enhanced imaging can be considered even more relevant.

Fourth, scan parameters varied at different MRI scanners during the included study period (2005–2016), leading to slightly different image quality and appearance: Contrast-to-noise ratio is significantly higher at 3 Tesla than at 1.5 Tesla[34] and high resolution isotropic 3D imaging is most accurate in depiction of small lesions. In that respect, contrast enhanced T1-weighting might have had an advantage over the other sequences as it is frequently acquired with the highest resolution.

Finally, type and dose of contrast media as well as time-to-acquisition differed between contrast-enhanced images. These factors have a well-known impact on lesion conspicuity: Signal-to-noise ratio increases with escalation of dose and time-delay between contrast media application and image acquisition[35–40].

Nonetheless, all scans were treated equally in the assessment, indicating that ceT1w imaging is superior to other sequences in the detection of MBM independently from contrast-media dependent parameters.

Our results suggest the optimization of contrast-enhanced MRI for even more precise detection of small MBM. In this study, mainly 1.5 Tesla MRI systems and 2D contrast-enhanced scans were used. It is well known that higher magnetic field strength and 3D MR images, such as MPRAGE and SPACE, are superior for detecting small lesions because of higher tumor-to-brain-contrast[34], less partial volume effect and less blood flow artifacts[14,41,42]. Several investigators have already reported the increase in lesion detection rate using SPACE imaging compared with MPRAGE imaging for evaluating brain metastases[43–45]. These studies have mentioned possible mechanisms, including higher contrast enhancement effect, greater magnetization transfer effect, and the effect of vascular signal suppression on SPACE[46–48].

## Conclusion

In patients with metastatic melanoma contrast-enhanced T1-weighting is the most sensitive MRI sequence for early detection of brain metastases. No other sequence including T2w-FLAIR or susceptibility-weighted imaging can fully replace the application of contrast media for highest sensitivity. It is therefore likely beneficial to optimize image quality and assessment of the contrast-enhanced sequence to precisely detect small MBM.

## References

[1] Owonikoko TK, Arbiser J, Zelnak A, Shu HG, Shim H, Robin AM, et al Current approaches to the treatment of metastatic brain tumours. Nat Rev Clin Oncol. 2014;11(4):203–22. doi: 10.1038/nrclinonc.2014.25 24569448 10.1038/nrclinonc.2014.25 PMC4041037

[2] Platta CS, Khuntia D, Mehta MP, Suh JH. Current Treatment Strategies for Brain Metastasis and Complications From Therapeutic Techniques NCF in Brain Metastasis. Am J Clin Oncol. 2010;33(4):398–407. doi: 10.1097/COC.0b013e318194f744 19675447 10.1097/COC.0b013e318194f744

[3] Amer M, Al-Sarraf M, Baker L, Vaitkevicius V. Malignant melanoma and central nervous system metastase, Incidence, Diagnosis, Treatment and Survival. Cancer. 1976;42(2):660–8. 10.1002/1097-0142(197808)42:2<660::aid-cncr2820420237>3.0.co;2-e 679158

[4] Davies MA, Liu P, McIntyre S, Kim KB, Papadopoulos N, Hwu WJ, et al Prognostic factors for survival in melanoma patients with brain metastases. Cancer. 2011;117(8):1687–96. doi: 10.1002/cncr.25634 20960525 10.1002/cncr.25634

[5] Budman DR, Camacho E, Wittes RE. The Current Causes of Death in Patients with Malignant Melanoma. Eur j Cancer. 1978;14:327–30. 648555 10.1016/0014-2964(78)90201-3

[6] Barnholtz-Sloan JS, Sloan AE, Davis FG, Vigneau FD, Lai P, Sawaya RE. Incidence proportions of brain metastases in patients diagnosed (1973 to 2001) in the Metropolitan Detroit Cancer Surveillance System. J Clin Oncol. 2004;22(14):2865–72. doi: 10.1200/JCO.2004.12.149 15254054 10.1200/JCO.2004.12.149

[7] McArthur GA, Chapman PB, Robert C, Larkin J, Haanen JB, Dummer R, et al Safety and efficacy of vemurafenib in BRAFV600E and BRAFV600K mutation-positive melanoma (BRIM-3): Extended follow-up of a phase 3, randomised, open-label study. Lancet Oncol. 2014;15:323–32. doi: 10.1016/S1470-2045(14)70012-9 24508103 10.1016/S1470-2045(14)70012-9 PMC4382632

[8] Spagnolo F, Picasso V, Lambertini M, Ottaviano V, Dozin B, Queirolo P. Survival of patients with metastatic melanoma and brain metastases in the era of MAP-kinase inhibitors and immunologic checkpoint blockade antibodies: A systematic review. Cancer Treat Rev [Internet]. 2016;45:38–45. Available from: http://dx.doi.org/10.1016/j.ctrv.2016.03.003 26975020 10.1016/j.ctrv.2016.03.003

[9] Long G V., Stroyakovskiy D, Gogas H, Levchenko E, De Braud F, Larkin J, et al Dabrafenib and trametinib versus dabrafenib and placebo for Val600 BRAF-mutant melanoma: A multicentre, double-blind, phase 3 randomised controlled trial. Lancet. 2015;386:444–51. doi: 10.1016/S0140-6736(15)60898-4 26037941 10.1016/S0140-6736(15)60898-4

[10] Chapman PB, Hauschild A, Robert C, Larkin JMG, Haanen JBAG, Ribas A, et al Updated overall survival (OS) results for BRIM-3, a phase III randomized, open-label, multicenter trial comparing BRAF inhibitor vemurafenib (vem) with dacarbazine (DTIC) in previously untreated patients with BRAFV600E-mutated melanoma. J Clin Oncol [Internet]. 2012 5 20;30(15_suppl):8502 Available from: http://ascopubs.org/doi/abs/10.1200/jco.2012.30.15_suppl.8502

[11] Force J, Salama A. First-line treatment of metastatic melanoma: role of Nivolumab. Immunotargets Ther. 2017;6:1–10. doi: 10.2147/ITT.S110479 28243579 10.2147/ITT.S110479 PMC5315343

[12] Hodi FS, O’Day SJ, McDermott DF, Weber RW, Sosman JA, Haanen JB, et al Improved Survival with Ipilimumab in Patients with Metastatic Melanoma. N Engl J Med. 2010;363(8):711–23. doi: 10.1056/NEJMoa1003466 20525992 10.1056/NEJMoa1003466 PMC3549297

[13] Fan B, Li M, Wang X, Xu Y, Li F, Zhang L, et al Diagnostic Value of Gadobutrol Versus Gadopentetate Dimeglumine in Enhanced MRI of Brain Metastases. J magn Reson Imaging. 2016; 10.1002/jmri.25491 27696616

[14] Kwak HS, Hwang S, Chung GH, Song JS, Choi EJ. Detection of small brain metastases at 3 T: Comparing the diagnostic performances of contrast-enhanced T1-weighted SPACE, MPRAGE, and 2D FLASH imaging. Clin Imaging. 2015;39:571–5. doi: 10.1016/j.clinimag.2015.02.010 25770904 10.1016/j.clinimag.2015.02.010

[15] Cohen-Inbar O, Xu Z, Dodson B, Rizvi T, Durst CR, Mukherjee S, et al Time-delayed contrast-enhanced MRI improves detection of brain metastases: a prospective validation of diagnostic yield. J Neurooncol. 2016;130:485–94. doi: 10.1007/s11060-016-2242-6 27568036 10.1007/s11060-016-2242-6

[16] Suh C, Jung S, Kim K, Pyo J. The detectability of brain metastases using contratst-enhanced spin-echo or gradient-echo images: a systematic review and meta-analysis. J Neurooncol. 2016;129(2):363–71. doi: 10.1007/s11060-016-2185-y 27324495 10.1007/s11060-016-2185-y

[17] Escott EJ. A variety of appearances of malignant melanoma in the head: a review. Radiographics. 2001;21(3):625–39. doi: 10.1148/radiographics.21.3.g01ma19625 11353111 10.1148/radiographics.21.3.g01ma19625

[18] Isiklar I, Leeds NE, Fuller G, Kumar A. Intracranial Metastatic Melanoma: Correlation between MR Imaging Characteristics and Melanin Content. Am J Roentgenol. 1995;165:1503–12. 7484597 10.2214/ajr.165.6.7484597

[19] Franceschi AM, Moschos SJ, Anders CK, Glaubiger S, Collichio FA, Lee CB, et al Use of Susceptibility-Weighted Imaging (SWI) in the Detection of Brain Hemorrhagic Metastases from Breast Cancer and Melanoma. J Comput Assist Tomogr. 2016;40(5):803–5. doi: 10.1097/RCT.0000000000000420 27636126 10.1097/RCT.0000000000000420 PMC5027959

[20] Gaviani P, Mullins ME, Braga TA, Hedley-Whyte ET, Halpern EF, Schaefer PS, et al Improved detection of metastatic melanoma by T2*-weighted imaging. Am J Neuroradiol. 2006;27(3):605–8. 16552002 PMC7976999

[21] Radbruch A, Weberling LD, Kieslich PJ, Eidel O, Burth S, Kickingereder P, et al High-signal intensity in the dentate nucleus and globus pallidus on unenhanced T1-weighted images: Evaluation of the macrocyclic Gadolinium-based contrast agent gadobutrol. Invest Radiol. 2015;50(12):805–10. doi: 10.1097/RLI.0000000000000227 26523910 10.1097/RLI.0000000000000227

[22] Radbruch A, Weberling LD, Kieslich PJ, Eidel O, Burth S, Kickingereder P, et al Gadolinium Retention in the Dentate Nucleus and Globus Pallidus Is Dependent on the Class of Contrast Agent. Radiology [Internet]. 2015;275(3):783–91. Available from: http://pubs.rsna.org/doi/10.1148/radiol.2015150337 25848905 10.1148/radiol.2015150337

[23] Ali S, Lee SK. Ipilimumab therapy for melanoma: A mimic of leptomeningeal metastases. Am J Neuroradiol. 2015;36(12):E69–70. doi: 10.3174/ajnr.A4581 26427830 10.3174/ajnr.A4581 PMC7964270

[24] Smirniotopoulos J, Murphy F, Rushing E, Rees J, Schroeder J. Patterns of Contrast Enhancement in the Brain and Meninges 1. Radiographics. 2007;27:525–51. doi: 10.1148/rg.272065155 17374867 10.1148/rg.272065155

[25] Eichler AF, Chung E, Kodack DP, Loeffler JS, Fukumura D, Jain RK. The biology of brain metastases-translation to new therapies. Nat Rev Clin Oncol. 2011;8:344–56. doi: 10.1038/nrclinonc.2011.58 21487419 10.1038/nrclinonc.2011.58 PMC3259742

[26] Zhang R-D, Price JE, Fujimaki T, Bucana CD, Fidler IJ. Differential Permeability of the Blood-Brain Barrier in Experimental Brain Metastases Produced by Human Neoplasms Implanted into Nude Mice. Am J Pathol. 1992;141(141):1115–24. 1443046 PMC1886664

[27] Gramsch C, Göricke SL, Behrens F, Zimmer L, Schadendorf D, Krasny A, et al Isolated cerebral susceptibility artefacts in patients with malignant melanoma: Metastasis or not? Eur Radiol. 2013;23:2622–7. doi: 10.1007/s00330-013-2857-3 23670820 10.1007/s00330-013-2857-3

[28] Beiderwellen K, Gomez B, Buchbender C, Hartung V, Poeppel TD, Nensa F, et al Depiction and characterization of liver lesions in whole body [18F]-FDG PET/MRI. Eur J Radiol. 2013;82(11):e669–75. doi: 10.1016/j.ejrad.2013.07.027 24011443 10.1016/j.ejrad.2013.07.027

[29] Costelloe CM, Madewell JE, Kundra V, Harrell RK, Bassett RL, Ma J. Conspicuity of bone metastases on fast Dixon-based multisequence whole-body MRI: Clinical utility per sequence. Magn Reson Imaging. 2013;31(5):669–75. doi: 10.1016/j.mri.2012.10.017 23290478 10.1016/j.mri.2012.10.017 PMC3648589

[30] Flechsig P, Zechmann CM, Schreiweis J, Kratochwil C, Rath D, Schwartz LH, et al Qualitative and quantitative image analysis of CT and MR imaging in patients with neuroendocrine liver metastases in comparison to 68Ga-DOTATOC PET. Eur J Radiol. 2015;84(8):1593–600. doi: 10.1016/j.ejrad.2015.04.009 25999064 10.1016/j.ejrad.2015.04.009

[31] Jeon JY, Choi JW, Roh HG, Moon WJ. Effect of imaging time in the magnetic resonance detection of intracerebral metastases using single dose gadobutrol. Korean J Radiol. 2014;15:145–50. doi: 10.3348/kjr.2014.15.1.145 24497805 10.3348/kjr.2014.15.1.145 PMC3909848

[32] Ohlmann-Knafo S, Tarnoki AD, Tarnoki DL, Pickuth D. MR Diagnosis of Bone Metastases at 1.5 T and 3 T: Can STIR Imaging Be Omitted? RoFo. 2015;187:924–32. doi: 10.1055/s-0035-1553207 26085176 10.1055/s-0035-1553207

[33] Radbruch A, Lutz K, Wiestler B, Baumer P, Heiland S, Wick W, et al Relevance of T2 signal changes in the assessment of progression of glioblastoma according to the Response Assessment in Neurooncology criteria. Neuro Oncol. 2011/12/08. 2012;14:222–9. doi: 10.1093/neuonc/nor200 22146386 10.1093/neuonc/nor200 PMC3266385

[34] Nöbauer-Huhmann I-M, Ba-Ssalamah A, Mlynarik V, Barth M, Schöggl A, Heimberger K, et al Magnetic Resonance Imaging Contrast Enhancement of Brain Tumors at 3 Tesla Versus 1.5 Tesla. Invest Radiol. 2002;37(3):114–9. 11882790 10.1097/00004424-200203000-00003

[35] Ba-Ssalamah A, Nöbauer-Huhmann IM, Pinker K, Schibany N, Prokesch R, Mehrain S, et al Effect of Contrast Dose and Field Strength in the Magnetic Resonance Detection of Brain Metastases. Invest Radiol. 2003;(38):415–22. 12821855 10.1097/01.RLI.0000067488.57101.bd

[36] Yuh WTC, Engelken JD, Muhonen MG, Mayr NA, Fisher DJ, Ehrhardt JC. Experience with High-Dose Gadolinium MR Imaging in the Evaluation of Brain Metastases. AJNR. 1992;13:335–45. 1595472 PMC8331800

[37] Yuh WTC, Tali ET, Nguyen HD, Simonson TM, Mayr NA, Fisher DJ. The effect of contrast dose, imaging time, and lesion size in the MR detection of intracerebral metastasis. Am J Neuroradiol. 1995;16:373–80. 7726087 PMC8338330

[38] Kim ES, Chang JH, Choi HS, Kim J, Lee SK. Diagnostic yield of double-dose gadobutrol in the detection of brain metastasis: Intraindividual comparison with double-dose gadopentetate dimeglumine. Am J Neuroradiol. 2010;31(6):1055–8. doi: 10.3174/ajnr.A2010 20110372 10.3174/ajnr.A2010 PMC7963932

[39] Anzalone N, Gerevini S, Scotti R, Vezzulli P, Picozzi P. Detection of cerebral metastases on magnetic resonance imaging: Intraindividual comparison of gadobutrol with gadopentetate dimeglumine. Acta radiol. 2009;50(8):933–40. doi: 10.1080/02841850903095385 19626475 10.1080/02841850903095385

[40] marina Kushnirsky, vinh Nguyen, Katz J, steinklein J, lisa rosen, craig warshall, et al Time-delayed contrast-enhanced MRI improves detection of brain metastases and apparent treatment volumes. J Neurosurg [Internet]. 2016;124(124):489–95. Available from: http://thejns.org/doi/abs/10.3171/2015.2.JNS141993 26361281 10.3171/2015.2.JNS141993

[41] Furutani K, Harada M, Mawlan M, Nishitani H. Difference in Enhancement Between Spin Echo and 3-Dimensional Fast Spoiled Gradient Recalled Acquisition in Steady State Magnetic Resonance Imaging of Brain Metastasis at 3-T Magnetic Resonance Imaging. J Comput Assist Tomogr. 2008;32:313–9. doi: 10.1097/RCT.0b013e318074fd9d 18379324 10.1097/RCT.0b013e318074fd9d

[42] Chen W, Wang L, Zhu W, Xia L, Qi J, Feng D, et al Multicontrast single-slab 3D MRI to detect cerebral metastasis. Am J Roentgenol. 2012;198:27–32. 22194476 10.2214/AJR.11.7030

[43] Komada T, Naganawa S, Ogawa H, Matsushima M, Kubota S, Kawai H, et al Contrast-enhanced MR Imaging of Metastatic Brain Tumor at 3 Tesla: Utility of T 1 -weighted SPACE Compared with 2D Spin Echo and 3D Gradient Echo Sequence. Magn Reson Med Sci. 2008;7(1):13–21. 18460844 10.2463/mrms.7.13

[44] Kato Y, Higano S, Tamura H, Mugikura S, Umetsu A, Murata T, et al Usefulness of Contrast-Enhanced T1-weighted Sampling Perfection with Application-Optimized Contrasts by Using Different Flip Angle Evolutions in Detection of Small Brain Metastasis at 3T MR Imaging: Comparison with Magnetization-Prepared Rapid Acquisition of Gradient Echo Imaging. Am J Neuroradiol. 2009;30:923–9. doi: 10.3174/ajnr.A1506 19213825 10.3174/ajnr.A1506 PMC7051676

[45] Reichert M, Morelli JN, Runge VM, Tao A, Von Ritschl R, Von Ritschl A, et al Contrast-Enhanced 3-Dimensional SPACE Versus MP-RAGE for the Detection of Brain Metastases Considerations With a 32-Channel Head Coil. Invest Radiol. 2013;48:55–60. doi: 10.1097/RLI.0b013e318277b1aa 23192164 10.1097/RLI.0b013e318277b1aa

[46] Chappell PM, Pelc NJ, Foo TKF, Glover GH, Haros SP, Enzmann DR. Comparison of lesion enhancement on spin-echo and gradient-echo images. Am J Neuroradiol. 1994;15:37–44. 8141064 PMC8332096

[47] Mugler Ill JP, Brookeman JR. Theoretical Analysis of Gadopentetate Dimeglumine Enhancement in X 1-weighted Imaging of the Brain: Comparison of Two-dimensional Spin-Echo and Three-dimensional Gradient-Echo Sequences’. J Magn Reson Imaging. 1993;3:761–9. 8400563 10.1002/jmri.1880030512

[48] Blüml S, Schad LR, Scharf J, Wenz F, Knopp M V., Lorenz WJ. A comparison of magnetization prepared 3D gradient-echo (MP-RAGE) sequences for imaging of intracranial lesions. Magn Reson Imaging. 1996;14:329–35. 8725198 10.1016/0730-725x(95)02095-b

